# Supplementary material for: Interpersonal brain synchronization during sensorimotor synchronization in people with different aerobic fitness levels: A fNIRS-based hyperscanning study
Source: PLoS One. 2025 Aug 18;20(8):e0329450. doi: 10.1371/journal.pone.0329450 (PMC12360550; doi:10.1371/journal.pone.0329450)
Supplement: Attachment — Submitted filename: Response to Reviewers.docx [file pone.0329450.s003.docx]

**Cover letter**

Dear Dr. Sun,

On behalf of all the contributing authors, I would like to express our sincere appreciation of your letter and reviewers’ constructive comments concerning our article entitled“Interpersonal brain synchronization during sensorimotor synchronization in people with different aerobic fitness levels: A fNIRS-Based hyperscanning study”(Manuscript ID PONE-D-24-44814).

We have considered the comments very carefully and have revised the paper accordingly.These comments are all valuable and helpful for improving our article. Point-by-point responses to the comments from the editor and the three reviewers can be found in the Responses to the Editor and Reviewers.

We confirm that all authors have reviewed and approved the content of the manuscript and agree to its submission to your journal. Information regarding funding sources and ethical statements has been disclosed in the manuscript as required. In addition, the funders were involved in the experimental design, data analysis, and the writing and revision of the manuscript.

Thanks again for your consideration of publishing our manuscript in your journal. We are looking forward to hearing from you soon.

Please let me know if any additional information is required.

Sincerely,

Shilin Wen, Ph.D., Professor
Capital University of Physical Education and Sports
Beijing 100091, People’s Republic of China
Email: 313811819@qq.com
Phone: +86-18001090696

**Response to the editor’s and reviewers’ comments**

**Dear Editor and Reviewers：**

We sincerely appreciate your thorough review of our manuscript. We are grateful for your constructive comments and suggestions, which have greatly helped us improve the quality of our manuscript.

We have carefully considered all the comments and have revised the manuscript accordingly. The revised content has been marked in red color.

Below, we provide a detailed point-by-point response to each comment.

**Journal Requirements:**

1. Please ensure that your manuscript meets PLOS ONE's style requirements, including those for file naming.

**Response**: Thank you for your reminder.

We have revised the format of the manuscript according to the requirements of the journal.

2. PLOS requires an ORCID iD for the corresponding author in Editorial Manager on papers submitted after December 6th, 2016. Please ensure that you have an ORCID iD and that it is validated in Editorial Manager. To do this, go to ‘Update my Information’ (in the upper left-hand corner of the main menu), and click on the Fetch/Validate link next to the ORCID field. This will take you to the ORCID site and allow you to create a new iD or authenticate a pre-existing iD in Editorial Manager..

**Response**: Thank you for your reminder.

We have verified the ORCID iD in the Editorial Manager system.

3.Please provide additional details regarding participant consent. In the ethics statement in the Methods and online submission information, please ensure that you have specified what type you obtained (for instance, written or verbal, and if verbal, how it was documented and witnessed). If your study included minors, state whether you obtained consent from parents or guardians. If the need for consent was waived by the ethics committee, please include this information.

**Response**: We have added an Ethics Statement in the Materials and Methods section of the manuscript. The specific content is as follows:

"This study was performed in accordance with the ethical standards specified in the latest Helsinki Declaration and was approved by the ethics committee of Capital University of Physical Education and Sports (2021A21). All participants signed the informed consent before the experiment and received certain remuneration after completing the experimental task."

4. Thank you for stating the following financial disclosure:

[Ministry of Education, Humanities and Social Sciences project (No. 22YJA890028].

Please state what role the funders took in the study. If the funders had no role, please state: ""The funders had no role in study design, data collection and analysis, decision to publish, or preparation of the manuscript.""

If this statement is not correct you must amend it as needed.

Please include this amended Role of Funder statement in your cover letter; we will change the online submission form on your behalf.

**Response**: Thank you for your reminder.

The funders were involved in the experimental design, data analysis, and the writing and revision of the manuscript. We have included this information in the cover letter.

5. All PLOS journals now require all data underlying the findings described in their manuscript to be freely available to other researchers, either 1. In a public repository, 2. Within the manuscript itself, or 3. Uploaded as supplementary information.

**Response**: Thank you for your reminder.

We have uploaded the data as supplementary information and have indicated this in the revised manuscript.

**Reviewer #1:**

Introduction

1. The term “sensorimotor synchronization” is misspelled (e.g., “Sensoriotor synchronization”). Please review the manuscript for consistency and correct this error throughout.

**Response**: Thank you for your helpful suggestions.

We have thoroughly checked and corrected all the spelling mistakes in the manuscript.

2. There is a lack of discussion on the relationship between aerobic exercise and SMS. How did the authors arrive at the conclusion “it is still unclear whether good aerobic fitness is associated with enhanced SMS ability and its potential mechanisms”? Why were these two variables linked? Is there a debate in the existing literature on this topic, or are theoretical concepts in this area yet to be verified? Please consider expanding on this point.

**Response**: Thank you for your helpful comments and suggestions.

We reviewed the published literature and did not find studies that specifically investigated the relationship between aerobic fitness and SMS ability. Therefore, we proposed that "it is still unclear whether good aerobic fitness is associated with enhanced SMS ability and its potential mechanisms." In addition, in the second and third paragraphs of the introduction, we explain how these two variables are linked. In the second paragraph of the introduction, we discuss the possible relationship between SMS and physical movement, providing examples of sports activities that can enhance SMS ability. In the third paragraph, we describe how aerobic exercise can improve brain structure and function and promote cognitive development, and note that it remains unclear whether aerobic exercise can improve SMS as a cognitive function.

3. The concept of ROI (Region of Interest) is not clearly defined. The manuscript only discusses the relationship between synchronized movement and the prefrontal or sensory-motor cortex, but it does not explain whether aerobic exercise and different rhythms also influence the prefrontal cortex. Further discussion on this is recommended.

**Response**: Thank you for your helpful comments and suggestions.

In the revised manuscript, we defined the concept of ROI in the Methods—Data Collection section as follows:

The prefrontal cortex was selected as the region of interest (ROI) for this study.

Since our study employs a hyperscanning paradigm for simultaneous multi-brain data collection, there are only a few hundred published experimental studies overall, and to date, no research has been found exploring the relationship between aerobic exercise, different rhythms, and the prefrontal cortex. So far, we have only identified two hyperscanning studies related to physical activities and the prefrontal cortex, which we introduced in the revised manuscript as follows:

Meanwhile, hyperscanning studies related to physical activities have found that both cycling and basketball are associated with enhanced inter-brain synchronization (IBS) in the prefrontal cortex, as well as improvements in specific task performance.

Regarding the variable of different rhythms, we provided the following introduction in the fourth paragraph of the Introduction:

In laboratory research on SMS, information processing methods are commonly employed, with finger-tapping being a typical task where individuals strive to synchronize their finger taps with an external rhythm. It is found that the performance of SMS tasks is directly influenced by the sequence interonset interval (IOI), with the optimal synchronization frequency occurring at approximately 2 Hz (around 400 ms–600 ms). At this frequency, performance differences among different populations are relatively small. However, as IOI increases, task performance declines and individual differences become more pronounced.

4. The study hypothesis is not included. Please add a research hypothesis grounded in theoretical or empirical evidence to address the research question.

**Response**: Thank you for your helpful comments and suggestions.

We have added the following hypothesis: Based on the beneficial effects of aerobic exercise on cognitive function and the enhancement of IBS in the prefrontal cortex induced by physical activity, the present study proposes the following hypothesis: Individuals with higher levels of aerobic fitness may exhibit superior SMS abilities, an advantage that becomes more pronounced as the IOI increases, possibly due to aerobic exercise enhancing task performance by facilitating IBS in the prefrontal cortex.

Methods

1. Why were long-distance runners specifically chosen for this study? What role do these subjects play in addressing the research question? Could other types of aerobic exercise groups also be applicable?

**Response**： Thanks for your comment.

There are two main reasons for selecting long-distance runners as participants in our study. First, from a training perspective, long-distance running is one of the most representative forms of aerobic exercise, which closely matches the aerobic fitness variable we are investigating. Second, our experiment was conducted during the COVID-19 pandemic, when it was difficult for other populations to engage in regular and systematic exercise. In contrast, the long-distance runners received organized training provided by the university, which helped them maintain a high level of aerobic fitness during the experimental period. As a result, these participants served as an effective means of controlling variables in addressing the research questions. Since most types of aerobic exercise possess periodic or rhythmic characteristics, we believe that our findings are applicable to most populations engaged in aerobic exercise.

2. Please clarify which irrelevant variables were controlled for in both the experimental and control groups. Did the control group assess aerobic fitness levels, and were subjects with expertise in rhythm control (e.g., pianists) or extensive aerobic exercise experience (e.g., regular gym-goers) excluded?

**Response**: Thank you for your helpful suggestions.

Except for the difference in exercise duration between the two groups, we tried to control for the consistency of other variables that might affect the experimental results. These included participants’ handedness, the level of familiarity within each matched pair, and musical instrument learning background. In addition, we ensured that members of the control group had less than one hour of exercise per week over the past three months, with no systematic gym training experience. In response to your comments, we have added the following content to the Participants section in the revised manuscript: None of the participants had received systematic musical instrument training. Members of the experimental group engaged in an average of no less than 6 hours of aerobic exercise per week in the past three months, while members of the control group self-reported an average of no more than 1 hour of exercise per week during the same period and had no history of systematic gym workouts.

3. The rationale for using 1000m run time as an indicator of aerobic fitness is unclear. Please provide supporting references.

**Response**: Thank you for your suggestions.

In the manuscript, we included two references after the sentence: “One week prior to the experiment, the participants' 1000-meter running times were recorded as a measure of their aerobic fitness level.” The first reference provides the following statement: “Field tests such as the 12-min run, 1.5-mile (2.4 km) run, and 1000-m run are frequently used to estimate cardiorespiratory fitness”^1^. The second reference supports the reliability of the 1000-meter run as an evaluation tool for aerobic fitness among elite soccer players^2^.

A maximal multistage 20-m shuttle run test to predict VO2 max

The concurrent validity and between-session reliability of a 1000m time trial The concurrent validity and between-session reliability of a 1000m time trial for the assessment of aerobic fitness in elite development soccer players

4. Please verify the consistency of channel placements across subjects. What is the variability in channel location data (MNI coordinates) between the two groups, and how do these differ?

**Response**: Thank you for your helpful suggestions.

We calculated the average MNI coordinates and corresponding Brodmann areas for both the experimental group and the control group. We found that the coordinates of each channel were essentially the same between the two groups, and all 17 channels corresponded to the same Brodmann areas. According to your suggestion, we have revised Table 1 in the manuscript. The revised table is shown below.

Table 1. The coordinates in MNI space and corresponding neuroanatomical labels for channels in the ROI.

| **Channel** | **MNI coordinates(E/C)** | | | **Brodman area** |
| --- | --- | --- | --- | --- |
|  | **X** | **Y** | **Z** |  |
| 1 | 27/28 | 25/24 | 61/62 | Dorsolateral prefrontal cortex |
| 2 | 5/6 | 27/26 | 64/65 | Dorsolateral prefrontal cortex |
| 3 | -17/-17 | 28/28 | 63/63 | Dorsolateral prefrontal cortex |
| 4 | 41/40 | 32/31 | 48/48 | Dorsolateral prefrontal cortex |
| 5 | 18/19 | 36/36 | 58/58 | Dorsolateral prefrontal cortex |
| 6 | -7/-6 | 40/40 | 57/57 | Dorsolateral prefrontal cortex |
| 7 | -26/-26 | 36/36 | 53/51 | Dorsolateral prefrontal cortex |
| 8 | 30/31 | 44/44 | 45/44 | Dorsolateral prefrontal cortex |
| 9 | 10/11 | 48/49 | 52/51 | Pre-Motor and Supplementary Motor Cortex |
| 10 | -14/-13 | 48/49 | 49/49 | Includes Frontal eye fields |
| 11 | 46/46 | 49/48 | 26/27 | Includes Frontal eye fields |
| 12 | 23/24 | 56/56 | 38/39 | Pre-Motor and Supplementary Motor Cortex |
| 13 | 0/0 | 56/56 | 39/39 | Pre-Motor and Supplementary Motor Cortex |
| 14 | -23/-23 | 56/57 | 36/36 | Pre-Motor and Supplementary Motor Cortex |
| 15 | 38/37 | 60/60 | 20/21 | Pre-Motor and Supplementary Motor Cortex |
| 16 | 14/15 | 67/66 | 29/28 | Includes Frontal eye fields |
| 17 | -10/-10 | 66/66 | 28/28 | Includes Frontal eye fields |

Note：E represents the experimental group, and C represents the control group.

5. In the data analysis section, the manuscript mentions that "HbO is more sensitive to blood flow changes." Please include supporting references for this claim.

**Response**: Thank you for your helpful suggestions.

We cited two references after this sentence to support this point^3,4^.

6. In Figure 2, is the data presented from a single subject or is it the average of all subjects?

**Response**: Thank you for your helpful suggestions.

This figure shows the HbO data from channel 2 of a paired participant. We described it in the manuscript as follows: "The coherence based on HbO signal from channel 2 (CH2) in a representative pair of the experimental group."

Results

1. Please include an analysis of the validity of the subjects’ behavioral responses, specifically comparing the MITI between the two groups and their corresponding control rhythms.

**Response**: Thank you for your helpful comments.

We provided the following introduction to the MITI in the Behavioral Data Processing and Analysis section of the Methods: Mean intertap interval(MITI): The MITI is used to assess whether the two groups of participants performed the task under the same conditions. It is obtained by calculating the mean interval between key presses for each participant within each trial. Previous research has found a strong correlation between the MITI and task performance. If there is a significant difference between the two groups, it indicates inconsistent task conditions between the groups, which can directly impact other experimental results.

In the Results section, there was no significant difference in MITI between the two groups, indicating that both groups completed the task with essentially the same tapping interval. We included the following statement in the Results section: This result indicates that both groups were under consistent task conditions across the three tasks, which adds credibility to the analysis of the task performance indicators.

2. Is there a correlation between the aerobic fitness level of the experimental group and IBS?

**Response**: Thank you for your helpful comments.

We conducted a correlation analysis between the experimental group's 1000 m run time and IBS during data processing, but did not find a significant correlation. We speculate that this may be because, although IBS is potentially related to aerobic fitness, the aerobic fitness levels of participants in the experimental group were relatively similar, and the relationship between IBS and aerobic fitness might not be reflected in such subtle differences. We believe that the significant enhancement of IBS observed only in the experimental group, but not in the control group, is already sufficient to support the main conclusions of our study. Therefore, we did not further analyze or discuss the relationship between aerobic fitness and IBS within the experimental group in the manuscript.

3. Does the relationship between MA/ITIV and IBS differ between the experimental and control groups?

**Response**: Thank you for your helpful comments.

We conducted Pearson correlation analyses between MA/ITIV and IBS for both groups during data analysis. The results showed that only the experimental group exhibited significant correlations, suggesting a difference in the correlations between the two groups. However, since significant IBS was observed only in the experimental group and not in the control group, this result is already sufficient to support the main conclusions of our study. We believe that, although comparing the correlations between the two groups could yield additional positive findings, such results are of limited significance. Therefore, we did not analyze the correlations between MA/ITIV and IBS in the control group in the manuscript.

Discussion

1. Under the 500ms condition, there were no differences in MA between the two groups, but significant differences in inter-brain synchrony were observed. What explains this discrepancy between the behavioral and brain activity results? Please discuss in detail.

**Response**: Thank you for your helpful suggestions.

Our results consist of three aspects: (1) there was no significant difference in MA between the two groups under the 500 ms condition; (2) there was a significant difference in IBS between the two groups in the 500 ms task; (3) in the experimental group, there was no significant correlation between IBS and MA in the 500 ms task.

Regarding the lack of a significant difference in MA between the two groups under the 500 ms condition, we discussed the following in the manuscript: The natural frequency of spontaneous human movement rhythm is approximately 2 Hz. This mechanism influences various abilities, such as attention, temporal expectation, and action coordination, resulting in the smallest MA at this frequency. Even individuals with specialized musical training do not exhibit differences from the general population at this frequency. However, as the task frequency decreases, performance differences between groups begin to emerge. For example, musicians start to show differences from the general population in IOI of 800 ms, and older adults exhibit significantly larger MA compared to young controls in IOI closer to 1 s (900 ms). The results of the current study are consistent with previous behavioral findings. The experimental group did not show better performance in IOI of 500 ms, but began to demonstrate an advantage in IOI of 1000 ms. Furthermore, this advantage tended to increase as the task difficulty level rose.

There was a significant difference in IBS between the two groups in the 500 ms task. In the revised manuscript, we discussed this as follows: We speculate that this result may be due to the relative simplicity of the finger-tapping task, which, for ordinary individuals, elicits a lower intensity of neural impulses. Therefore, no significant IBS was observed in the control group. In contrast, higher aerobic fitness may have a beneficial effect on neural plasticity, enhancing neural impulses during the task. As a result, significant IBS was observed in the experimental group.

Regarding the lack of a significant correlation between IBS and MA in the experimental group during the 500 ms task, we discussed the following in the revised manuscript：However, in the IOI of 500 ms, the experimental group's MA did not significantly correlate with the IBS. This may be because the task was closer to the natural spontaneous rhythm, and the unconscious components during the task had a greater impact on synchronization performance, which may have masked the relationship between MA and IBS.

**Reviewer #2:**

There is only one point that the authors should consider

The manuscript title names as "interpersonal synchronization" the correspondence between physiological variables of two individuals responding to the same external sytimuli. This is not interpersonal synchronization,that is usually referred to the similar behavioral and physiological activities observable in two persons looking at each other and reflecting each other.

Thus, I suggest to change the title, erasing the "interpersonal synchronization" , and focus the descrpition of results and discussion on the effect of fitness on the individual ability to follow the external stimuli

**Response**: Thank you for your helpful suggestions.

I believe there may be a misunderstanding regarding our title. Our title does not include the term "interpersonal synchronization," but rather "Interpersonal brain synchronization (IBS)," which refers to the phenomenon where the brain activities of two or more individuals show a certain degree of similarity or synchronization during social interaction.

Regarding the situation you mentioned—"the correspondence between physiological variables of two individuals responding to the same external stimuli"—we refer to this as sensorimotor synchronization(SMS) in our title and manuscript. SMS refers to the process in which individuals coordinate their own actions in time with perceivable external events and rhythms.

As for your suggestions on the Results and Discussion sections, we have made corresponding revisions as per your advice, making these sections focus more on "the effect of physical fitness on an individual's ability to respond to external stimuli." The revised content has been highlighted in red in the manuscript.

Reviewer #3: General comments.

1) Authors use to much abbreviations, which does not facilitate the understanding of the text. Perhaps it should be convenient and if authors agree, the use of a table with all the abbreviations used in the text at the end of the manuscript. This is one of the difficulties in following authors ‘arguments.

**Response**: Thank you for your helpful suggestions.

According to your suggestion and with reference to papers already published in this journal, we have added a list of abbreviations in the manuscript. The table is as follows:

**SMS**

sensorimotor synchronization

**fNIRS**

functional near-infrared spectroscopy

**IBS**

interpersonal brain synchronization

**rSFG**

right superior frontal gyrus

**SMT**

synchronized metronome training

**IOI**

interonset interval

**ITI**

intertap interval

**PCG**

phase correction gain

**HbO**

oxyhemoglobin

**HbR**

deoxyhemoglobin

**HbT**

total hemoglobin

**ROI**

region of interest

**CHs**

channels

**MITI**

mean intertap interval

**MA**

mean asynchrony

**ITIV**

intertap interval variability

2) Discrepant definitions between declarations of variables in Methods and their presentations in the figures (“Synchrony Performance, Average asynchrony identified as “AS”, pp. 16, and in Figure 4, as “MA”; Inter-Brain synchronization identified as “IBS”, pp. 9, and Interpersonal Brain Synchronization with the same abbreviation [IBS] Figure 6). Declarations of variables with the same meaning or the same abbreviations should be consistent along the paper to avoid confusing the reader.

**Response**: Thank you for your helpful suggestions.

Due to my oversight, the same variable was not expressed consistently throughout the manuscript. We have carefully re-examined the manuscript and have replaced all instances of “Average asynchrony (AS)” with “mean asynchrony (MA),” and all occurrences of “Inter-Brain synchronization” with “Interpersonal Brain Synchronization” to ensure consistency.

3) Paper is focalized into Interpersonal Brain Synchronization and Sensorimotor Synchronization. However, no explicit declaration about how these variables are experimentally measured and its meaning when values of variables changes in its range is given in Material and Methods. It will be very helpful to general readers if authors give an additional complementary explanation.

**Response**: Thank you for your helpful suggestions.

For sensorimotor synchronization, we recorded four behavioral indicators and introduced the calculation methods and significance of each indicator in the Behavioral Data Processing and Analysis section of the manuscript. The content is as follows:

Mean intertap interval(MITI): The MITI is used to assess whether the two groups of participants performed the task under the same conditions. It is obtained by calculating the mean interval between key presses for each participant within each trial. Previous research has found a strong correlation between the MITI and task performance. If there is a significant difference between the two groups, it indicates inconsistent task conditions between the groups, which can directly impact other experimental results^5^.

Synchrony Performance: Mean Asynchrony(MA) is used to evaluate the performance of the SMS task. It is obtained by subtracting the key press time point of one participant from the corresponding key press time point of another participant, and then calculating the mean of the absolute values of the asynchronies. In the same condition types, a smaller MA represents stronger synchrony ability.

Correction Ability: The PCG is selected as an indicator to evaluate the correction ability. PCG represents the extent to which participants adjust the timing of their next key press based on the previous asynchrony. The closer the average value of PCG is to 1 for each pair of participants, the stronger the correction ability of the synchrony system. The calculation method adopts the bivariate Generalized Least Squares (bGLS) model.

Stability: intertap interval variability(ITIV) is used as an indicator to assess stability. In the same condition types, a smaller ITIV represents more stable time control ability. It is obtained by calculating the standard deviation of the ITI for each participant within each trial.

However, it may have caused confusion because we did not provide a general overview in that section. In the revised manuscript, we have added a general description at the beginning of the Behavioral Data Processing and Analysis section:

"This study analyzed four behavioral indicators related to sensorimotor synchronization. The calculation methods and significance of each indicator are as follows:"

For Interpersonal Brain Synchronization, we provided the following description in the fNIRS Data Analysis section of the revised manuscript:For each pair of CHs between two participants, the average interpersonal brain synchronization enhancement, or IBS, was calculated in task period and resting period. We defined IBS as the task-related interpersonal brain synchronization enhancement (i.e., mean(block) - baseline). The larger the value, the stronger the task-related enhancement of IBS.Then，we converted IBS values to Fisher z-statistics and performed a one-sample t-test against zero across all channels, applying false discovery rate (FDR) correction (p < 0.05).

4) There is some technically problem with the correlation-regression analysis performed in figures 4-7.

In page 8, it is declared that MA in the IOI= 1000 ms is 79.50 ± 19.1. This means that for 79.5 ms the most higher value is 98.6 ms, and the most lower value is 60.4 ms. Statistically, this range represent one population with a mean value of 79.50 ms. In Figure 6 B, as an example, the range of values for the independent variable (MA), 60.4 -98.6 ms, represent just only one value for the dependent variable IBS. Thus, the cluster of points of IBS between 60.4 – 98.6 ms are not really different covariant values of MA. They represent only an estimate of the covariant IBS, when MA = 79.50. This is so, because authors decided to use the statistical Model 2 for the regression analysis. My suggestion is that the correlation-regression analysis should be eliminated, since authors have enough evidence supporting the hypothesis that the condition of aerobic fitness is influencing the parameters under study. A similar comment for the rest is valid.

**Response**: Thank you for your helpful suggestions.

I apologize if my previous explanation was unclear and led to any misunderstanding of the data. Taking MA in the IOI = 1000 ms as an example, the data value 79.50 ± 19.1 represents the mean ± standard deviation; in this case, 19.1 refers to the range of one standard deviation, and does not indicate that the MA ranges from 60.4 to 98.6 ms.

For the IOI = 1000 ms task, each pair of participants has one MA value and one IBS value. As shown in the figure, the MA values for the 23 pairs in the experimental group under this condition roughly range from 50 to 125, while the IBS values range from about -0.06 to 0.08. For each pair, we performed a Pearson correlation analysis between their own MA and IBS values. The results of this correlation analysis are presented in the figure, indicating a significant negative correlation between MA and IBS (i.e., the greater the IBS, the lower the MA value). From a behavioral perspective, a smaller MA value denotes better task performance.

Therefore, this result demonstrates a positive relationship between IBS and task performance, which provides strong support for our study. An important aspect of hyperscanning research is to explore the relationship between neural indicators and behavioral performance through correlation analysis, establishing the link between brain activity and behavior. Thus, we believe it is necessary to retain the results of this correlation analysis.

**References:**

1	Leger, L. A. & Lambert, J. A maximal multistage 20-m shuttle run test to predict O2 max. *European journal of applied physiology and occupational physiology* **49**, 1-12 (1982).

2	Clancy, C., Green, P., Curnyn, S., Donaldson, E. & Ring, N. The concurrent validity and between-session reliability of a 1000m time trial for the assessment of aerobic fitness in elite development soccer players. *Sport Performance and Science Reports* **92**, 1-3 (2020).

3	Hoshi, Y. Functional near‐infrared optical imaging: Utility and limitations in human brain mapping. *psychophysiology* **40**, 511-520 (2003).

4	Hoshi, Y. Functional near-infrared spectroscopy: current status and future prospects. *Journal of biomedical optics* **12**, 062106-062106-062109 (2007).

5	Repp, B. H. Sensorimotor synchronization: A review of the tapping literature. *Psychonomic Bulletin & Review* **12**, 969-992, doi:10.3758/BF03206433 (2005).
