# Supplementary material for: Interpersonal brain synchronization during sensorimotor synchronization in people with different aerobic fitness levels: A fNIRS-based hyperscanning study
Source: PLoS One. 2025 Aug 18;20(8):e0329450. doi: 10.1371/journal.pone.0329450 (PMC12360550; doi:10.1371/journal.pone.0329450)
Supplement: Attachment — Submitted filename: Response_to_Reviewers_auresp_2.docx [file pone.0329450.s004.docx]

**Response to the reviewer’s comments**

**Dear Reviewers：**

We sincerely appreciate your thorough review of our manuscript. We are grateful for your constructive comments and suggestions, which have greatly helped us improve the quality of our manuscript.

We have carefully considered all the comments and have revised the manuscript accordingly. The revised content has been marked in red color.

Below is our detailed response to your comments.

**Reviewer #1:**

Reviewer #1: Regarding the introduction section, it seems the author may have misunderstood my original question.

The second paragraph of the introduction emphasizes two main points. First, that sensorimotor synchronization (SMS) training can enhance motor performance. Second, that motor experience influences SMS. Based on these two points, the author concludes that there is a bidirectional relationship between SMS and physical activity. However, the focus of the present study is aerobic fitness. What is the relationship between aerobic fitness and physical activity? Can aerobic fitness be equated with physical activity? The core concepts in these two paragraphs are inconsistent.

Moreover, based on the main research content of this study, the independent variables are aerobic fitness level and IOI (inter-onset interval, i.e., rhythm), while the dependent variables include measures of synchronization performance and neural signals reflecting inter-brain synchronization. Therefore, the introduction should primarily address how the independent variables influence the dependent variables and their possible neural underpinnings—in other words, how aerobic fitness and rhythm affect synchronization, particularly the underlying inter-brain mechanisms.

Specifically, the emphasis should be on how aerobic fitness modulates SMS under different rhythmic conditions. For example, rhythm affects the difficulty of synchronization. According to the forward prediction model, individuals form predictions in advance about the sensory consequences of their actions and compare these predictions with actual sensory feedback to make corrections. Aerobic exercise continuously trains this “prediction–execution–feedback–adjustment” loop, thereby enhancing motor performance. Thus, the higher the individual’s aerobic fitness level, the more robust their forward prediction model becomes, leading to better motor performance across varying levels of difficulty.

Additionally, based on dynamic systems theory, aerobic exercise strengthens the dynamic regulation of perception–action–environment coupling, which in turn enhances synchronization performance.

Furthermore, the discussion of neuroimaging in the introduction is insufficient. For instance, in the third paragraph, what specific brain regions are affected by aerobic exercise-induced plasticity? What roles do these regions play in SMS? The second-to-last paragraph of the introduction only mentions that the prefrontal cortex is involved in SMS, but what is its relationship with aerobic exercise?

A more coherent line of reasoning might be: aerobic exercise enhances the forward prediction model, which is associated with the prefrontal cortex and premotor cortex. SMS under different IOI conditions involves the prefrontal cortex and sensorimotor cortex. Both mechanisms implicate the prefrontal and sensorimotor cortices. The more synchronized individuals’ behavioral performance is, the more accurate their prediction of each other’s movements, which is reflected in more synchronized neural activity—in other words, higher inter-brain synchrony.

**Response**: Thank you for your helpful suggestions.

We have carefully revised the introduction section of the manuscript in accordance with your suggestions. The specific modifications are as follows:

(1) We have removed the original second paragraph from the manuscript.

(2) In accordance with your suggestions, we have incorporated a discussion of neuroimaging findings into the revised second paragraph (which corresponds to the third paragraph in the original manuscript). Specifically, we have included examples illustrating the enhancing effects of aerobic exercise on the prefrontal cortex, and further clarified the role of the prefrontal cortex in SMS tasks. The revised paragraph now proceeds as follows: it first introduces the concept of aerobic fitness and its facilitating effects on various cognitive functions. It then explains, from a neuroimaging perspective, that aerobic exercise increases the activation level of the prefrontal cortex. Next, it emphasizes the critical role of the prefrontal cortex in SMS tasks. Finally, it points out the current limitation in the literature, namely the lack of studies directly examining the relationship between aerobic fitness and SMS ability. The revised content is as follows:

Aerobic fitness refers to the capacity to sustain muscular power or speed over extended periods, and is also commonly termed cardiovascular fitness. It is well established that superior aerobic fitness can be achieved through regular physical exercise. A substantial body of research has demonstrated that consistent engagement in physical activity, or possessing a high level of aerobic fitness, not only contributes to overall physical health but also increases the production of beneficial neurochemicals during exercise. This, in turn, enhances neural circuit efficiency, induces structural and functional changes in the brain, and yields long-lasting, positive effects on a range of cognitive functions. Evidence from neuroimaging studies indicates that a key brain region involved in these effects is the prefrontal cortex. For example, For example, studies have found that aerobic exercise can increase the volume and density of the prefrontal cortex, thereby enhancing memory function. In another study, children aged 8–9 years engaged in one hour of physical activity daily after school for nine months. Functional MRI was used to monitor cerebral blood flow during a flanker task that assessed attention and inhibitory control. Results revealed that these children exhibited behavioral performance and prefrontal activation patterns comparable to those of college students. In the context of SMS tasks, the prefrontal cortex operates as part of the “cognitively controlled timing” system and plays a critical role in task execution. However, it remains unclear whether optimal aerobic fitness can enhance SMS abilities, and the underlying neural mechanisms require further investigation.

(3) In accordance with your suggestions, we have revised the third paragraph (originally the fourth paragraph) to include a discussion of how aerobic exercise modulates the “prediction–execution–feedback–adjustment” loop within the forward prediction model, as well as the way in which aerobic exercise enhances the dynamic regulation of perception–action–environment coupling from the perspective of dynamic systems theory. In the revised manuscript, we have established a clearer connection between aerobic fitness and SMS performance under varying IOI conditions. The revised content is as follows:

In laboratory research on SMS, information processing methods are commonly employed, with the finger-tapping being a typical task where individuals strive to synchronize their finger taps with an external rhythm. It is found that the performance of SMS tasks is directly influenced by the sequence interonset interval (IOI) , with the optimal synchronization frequency occurring at approximately 2 Hz (around 400 ms-600 ms). At this frequency, performance differences among different populations are relatively small. However, with IOI extending, task performance declined and difference among individual increased.The forward prediction model offers a plausible explanation for these findings. According to this model, individuals generate predictions in advance regarding the sensory consequences of their actions and subsequently compare these predictions with actual sensory feedback to make corrections—this forms the so-called "prediction– execution– feedback– adjustment" loop. The key components of this loop are prediction and adjustment. As the IOI increases, the difficulty in generating accurate predictions becomes substantially greater, thereby leading to increased individual variability. Aerobic exercise has been shown to promote structural and functional plasticity in brain regions intimately involved in motor control, such as the prefrontal cortex. This enhancement boosts the efficiency of information integration and processing within these regions, thereby strengthening the predictive and adaptive elements of the aforementioned loop. Furthermore, in line with dynamic systems theory, aerobic exercise augments the dynamic regulation of the perception– action– environment coupling. Thus, we hypothesize that individuals with higher levels of aerobic fitness may demonstrate superior performance in SMS tasks.
